# Domain-Level Distribution of Pathogenic BRCA1/2 Somatic Mutations Shows No Evidence of Large Subtype-Specific Enrichment in Breast Cancer: A Three-Cohort Analysis Supporting Broad BRCA Testing

**DOI:** 10.3390/genes17060693

**Published:** 2026-06-13

**Authors:** Elif Sertesen Çamöz, Fatih Yıldız, Mutlu Dogan, Yunus Kasım Terzi, Zerrin Yılmaz Çelik

**Affiliations:** 1 Department of Medical Oncology, Dr. Abdurrahman Yurtaslan Ankara Oncology Training and Research Hospital, University of Health Sciences, Ankara 06200, Turkey; fatih.yildiz@sbu.edu.tr (F.Y.); mutlu.dogan@sbu.edu.tr (M.D.); 2 Department of Medical Genetics, Başkent University, Ankara 06490, Turkey; ykterzi@baskent.edu.tr (Y.K.T.); zerriny@baskent.edu.tr (Z.Y.Ç.)

**Keywords:** *BRCA1*, *BRCA2*, somatic mutation, breast cancer, triple-negative, hormone receptor, protein domain, PARP inhibitor, variant classification, pathogenicity

## Abstract

**Background**: Pathogenic *BRCA1* and *BRCA2* mutations confer a homologous recombination deficiency that underlies PARP inhibitor sensitivity. While *BRCA1* mutation carriers more frequently develop triple-negative breast cancer (TNBC) and *BRCA2* carriers hormone receptor-positive (HR+) disease, whether the specific protein domain harboring a pathogenic somatic mutation differs systematically between breast cancer subtypes remains uncertain. Apparent domain enrichment in earlier unfiltered analyses may be confounded by missense variants of uncertain significance (VUSs), which lack clinical actionability. **Methods**: We assembled three independent breast cancer cohorts via cBioPortal: TCGA-BRCA (brca_tcga_pub2015), METABRIC (brca_metabric), and MSK-CHORD (msk_chord_2024). All somatic *BRCA1/2* mutations were mapped to UniProt-annotated functional domains and to Rebbeck-defined breast/ovarian cancer cluster regions (BCCR/OCCR). Per ENIGMA/ACMG guidance, pathogenic mutations (nonsense, frameshift, and canonical splice site) were analyzed inferentially, while missense and in-frame variants—predominantly VUSs—were only reported descriptively. Fisher’s exact tests with Benjamini–Hochberg FDR correction were applied across domain × subtype contingencies. Cohort heterogeneity was assessed via Cochran’s Q and I^2^ statistics; pooled effect estimates were computed using inverse-variance fixed-effects meta-analysis. **Results**: A total of 394 somatic *BRCA1/2* mutations were identified across the three cohorts (*BRCA1 n* = 166; *BRCA2 n* = 228), of which 147 (37.3%) met pathogenic criteria. Among 131 pathogenic mutations in HR+/HER2− or TNBC subtypes, 84 (64.1%) occurred in HR+/HER2− disease and 47 (35.9%) in TNBC. Domain-level distributions did not differ significantly between subtypes for any *BRCA1* domain (BRCT: TNBC 20.0% vs. HR+ 18.8%, OR = 1.08, 95% CI 0.31–3.78, and FDR-adjusted *p* = 1.00) or *BRCA2* domain (DBD: TNBC 17.6% vs. HR+ 30.8%, OR = 0.48, and FDR-adjusted *p* = 1.00). Cluster-region analyses (nine Rebbeck BCCR/OCCRs) similarly showed no significant enrichment. Post hoc power analysis indicated that the study could only reliably detect large effects (OR ≥ ~3.0 for the principal BRCT contrast), and formal equivalence testing (TOST) demonstrated equivalence within a prespecified ±20% margin for *BRCA1* BRCT (TOST *p* = 0.031). Heterogeneity across cohorts was minimal (Cochran’s Q = 0.62, I^2^ = 0.0%). Descriptive analyses of VUSs suggested the apparent enrichment of BRCA1 BRCT-localized missense variants in TNBC (31.8% vs. 17.9% in HR+), but this signal did not extend to pathogenic mutations. **Conclusions**: Within the statistical power available, our three-cohort analysis shows no evidence of large subtype-specific enrichment of pathogenic *BRCA1/2* somatic mutations across protein domains or cluster regions; small to moderate effects cannot be excluded. Notably, the majority (64%) of pathogenic mutations occurred in HR+/HER2− disease, underscoring that *BRCA1/2* testing should not be deprioritized in non-TNBC subtypes. The apparent BRCT enrichment observed in earlier unfiltered analyses appears to be driven by VUSs rather than pathogenic variants, highlighting the methodological necessity of pathogenicity filtering for clinically actionable inference. These findings provide cohort-scale supportive evidence for emerging clinical guidelines that recommend broader *BRCA1/2* testing across breast cancer subtypes.

## 1. Introduction

*BRCA1* and *BRCA2* are tumor suppressor genes encoding proteins that are essential mediators of the homologous recombination (HR) repair of DNA double-strand breaks [1,2]. The loss of either protein produces a state of HR deficiency (HRD) that is synthetic lethal with poly(ADP-ribose) polymerase (PARP) inhibition, providing the molecular rationale for the clinical use of PARP inhibitors in BRCA-mutant breast and ovarian cancers [3,4]. Germline *BRCA1/2* pathogenic variants account for the majority of hereditary breast and ovarian cancer, and somatic *BRCA1/2* mutations are increasingly recognized as clinically actionable in sporadic disease, particularly with the expanding labeling of PARP inhibitors and the inclusion of tumor BRCA testing in major breast cancer treatment guidelines [5,6,7].

Breast cancer is a molecularly heterogeneous disease classified by hormone receptor (HR; estrogen receptor and/or progesterone receptor) and human epidermal growth factor receptor 2 (HER2) status into four major immunohistochemical subtypes: HR+/HER2−, HR+/HER2+, HR−/HER2+, and triple-negative breast cancer (TNBC; HR−/HER2−) [8]. *BRCA1* pathogenic variants preferentially associate with TNBC, while *BRCA2* carriers more frequently develop HR+ disease [9,10]. This association has fostered a clinical perception that *BRCA1/2* testing is most informative in TNBC, which is in turn reflected in some testing practices that prioritize TNBC over hormone receptor-positive disease [11]. However, large registry studies indicate that pathogenic *BRCA1/2* variants are present in a clinically meaningful proportion of HR+/HER2− breast cancers, and contemporary guidelines have moved toward broader testing eligibility independent of subtype [12,13].

Beyond the binary presence or absence of a BRCA1/2 mutation, an intriguing but incompletely answered question is whether the intragenic location of the mutation—the specific functional protein domain affected—influences the resulting tumor phenotype. BRCA1 is organized into the N-terminal RING domain, which mediates BARD1 heterodimerization and E3 ubiquitin ligase activity; a large central region encompassing exon 11; and the C-terminal BRCT domain, which mediates the phosphoprotein-dependent recruitment of HR effectors [14,15]. BRCA2 contains the N-terminal transactivation region, eight central BRC repeats that recruit and stabilize RAD51, and a C-terminal DNA-binding domain (DBD) comprising helical and oligonucleotide-binding fold subdomains [16,17]. Rebbeck and colleagues, in a landmark CIMBA-based analysis of germline BRCA1/2 carriers, identified breast and ovarian cancer cluster regions (BCCR/OCCR) within both genes, demonstrating that the relative breast-versus-ovarian cancer risk depends on mutation location [18]. Whether analogous subtype-specific patterns exist for somatic BRCA1/2 mutations within breast cancer subtypes has been the subject of small exploratory analyses [19], and we previously hypothesized that the BRCA1 BRCT domain might be preferentially mutated in TNBC.

A critical methodological consideration is that not all *BRCA1/2* mutations carry equivalent functional or clinical significance. Truncating mutations (nonsense, frameshift, and canonical splice site) are reliably loss-of-function and constitute the canonical pathogenic class invoked for PARP inhibitor decision-making [20]. Missense and in-frame variants, in contrast, are predominantly classified as variants of uncertain significance (VUSs) and are not actionable in clinical practice per ASCO and ACMG guidance [21,22]. This distinction is essential when analyzing domain-level patterns, because missense passenger variants may accumulate within protein domains in ways that do not reflect functional pathology [23]. In this study, we revisited the question of subtype-specific *BRCA1/2* domain enrichment using three large, independent breast cancer cohorts—TCGA-BRCA, METABRIC, and the recently published MSK-CHORD (Nature 2024) cohort, which together provide 394 somatic *BRCA1/2* mutations. We applied prespecified pathogenicity filtering, restricted inferential analyses to pathogenic (truncating) mutations, and reported VUS distributions descriptively. We additionally examined Rebbeck-defined BCCR/OCCR cluster regions in our somatic dataset. The primary objective was to determine whether pathogenic *BRCA1/2* somatic mutations show subtype-specific intragenic patterns of clinical relevance, and the secondary objective was to assess whether earlier reports of domain enrichment based on unfiltered mutation analyses might reflect VUSs rather than pathogenic variants.

## 2. Materials and Methods

### 2.1. Data Sources and Cohort Selection

Three large, publicly accessible breast cancer cohorts with annotated somatic *BRCA1/2* mutation data and clinical receptor status were selected for analysis through the cBioPortal for Cancer Genomics (https://www.cbioportal.org, accessed on 15 May 2026) [24,25]. The cohorts were chosen to provide complementary methodological and clinical coverage. TCGA-BRCA (study ID: brca_tcga_pub2015; *n* = 817) [26] contributes whole-exome sequencing with comprehensive immunohistochemistry-confirmed receptor profiling on predominantly primary tumors. METABRIC (study ID: brca_metabric; *n* = 2509) [27,28] contributes targeted sequencing with mature clinicopathologic annotation and the largest single-cohort breast cancer follow-up. MSK-CHORD (study ID: msk_chord_2024; *n* = 25,040) [29], the largest clinical-grade tumor sequencing dataset published to date, contributes contemporary MSK-IMPACT targeted sequencing with NLP-extracted hormone receptor and HER2 status, providing substantial representation of metastatic and advanced disease. These three cohorts were specifically selected over alternative MSK-IMPACT releases (e.g., breast_msk_2018) because MSK-CHORD supersedes prior MSK breast cancer datasets and provides the largest non-overlapping validation cohort.

### 2.2. Patient Selection and Subtype Classification

From each cohort, patients with one or more somatic BRCA1 and/or BRCA2 mutations were identified. Tumor receptor status was obtained from the corresponding clinical data files. HR status was defined as positive if estrogen receptor (ER) or progesterone receptor (PR) was reported as positive by immunohistochemistry and negative if both were reported as negative; HER2 status was defined per the cohort-reported overall HER2 result (IHC and FISH, harmonized). Patients were classified into four molecular subtypes: HR+/HER2−, HR+/HER2+, HR−/HER2+, and triple-negative (HR−/HER2−). The primary subtype comparison was prespecified as HR+/HER2− versus TNBC, consistent with the two clinically distinct extremes of receptor profiling. METABRIC samples lacking complete receptor information were excluded from inferential analyses but reported in descriptive cohort tables.

### 2.3. Mutation Retrieval, Quality Control, and Annotation

Somatic *BRCA1* (Entrez Gene ID 672, RefSeq NM_007294, UniProt P38398) and *BRCA2* (Entrez Gene ID 675, RefSeq NM_000059, UniProt P51587) mutations were retrieved via the cBioPortal REST API. For each cohort, mutation calling, alignment, and variant filtering had been performed by the original consortia using cohort-specific pipelines: TCGA-BRCA used the MC3 multi-caller pipeline with consensus filtering [30] and matched normal blood/saliva for somatic calling; METABRIC used targeted Agilent SureSelect panel sequencing (covering 173 cancer-related genes including *BRCA1/2*) with the bcgsc pipeline; MSK-CHORD used the MSK-IMPACT clinical-grade targeted panel (468 genes including *BRCA1/2*) with FDA-authorized variant calling. Importantly, MSK-IMPACT is performed at MSKCC as a matched tumor/normal test, with patient blood serving as the germline reference, allowing for the confident distinction of somatic from germline variants; only somatic calls were used in our analyses [31,32]. All three platforms provide full coding-region coverage of *BRCA1* and *BRCA2*. However, the three cohorts are institutionally and temporally independent (TCGA: NCI consortium; METABRIC: Cambridge/Vancouver pre-2010; and MSK-CHORD: MSKCC 2014–2023), and we are not aware of any patient overlap between them. The three sequencing platforms may differ in ascertainment sensitivity for specific variant classes—particularly indels, splice variants beyond canonical ±1/±2 positions, and large structural rearrangements—which we acknowledge as a potential source of differential ascertainment beyond cohort stage composition. Only somatic mutations from the curated, study-released mutation tables were used; we did not perform additional filtering beyond the cohort-level quality control. For each mutation, the following fields were extracted: protein change (HGVS p.), mutation type, protein position (UniProt-based amino acid coordinates), and variant allele frequency where available.

### 2.4. Variant Classification: Pathogenic Versus VUS

Per ENIGMA consortium and ACMG/AMP guidelines, *BRCA1/2* variants were classified into two analytic categories based on mutation type. Pathogenic (truncating) mutations comprised nonsense, frameshift insertion or deletion, and canonical splice-site variants—the classes for which loss-of-function pathogenicity can be inferred directly from the genetic code without additional functional evidence and which constitute the canonical actionable variant set for PARP inhibitor decision-making [20,22]. Missense, in-frame insertion, and in-frame deletion variants were classified as variants of uncertain significance (VUSs) for the purposes of this analysis, reflecting the well-established observation that the majority of *BRCA1/2* missense variants in tumor sequencing datasets remain unclassified or have variable functional consequences [33,34]. This classification scheme was prespecified prior to analysis. Pathogenic mutations constituted the primary analytic dataset for all inferential analyses. VUSs were only reported descriptively and were explicitly excluded from inferential testing, consistent with the ASCO position statement that VUSs should not factor into clinical decision-making.

### 2.5. Protein Domain and Cluster-Region Annotation

Each mutation was mapped to its annotated protein domain using UniProt coordinates. BRCA1 domains were defined as RING (residues 1–109), inter-domain region (110–1645), BRCT (1646–1855), and beyond-BRCT (1856–1863). BRCA2 domains were defined as N-terminal region (1–1001), BRC repeats (1002–2085), inter-DBD region (2086–2478), DBD (2479–3186), and C-terminal region (3187–3418) [14,15,16,17]. In addition, each mutation was annotated according to the Rebbeck et al. (2015) breast cancer cluster regions (BCCRs) and ovarian cancer cluster regions (OCCRs), with cDNA-based boundaries converted to amino acid coordinates [18]: for BRCA1, BCCR1 (aa 60–169), OCCR (aa 460–1354), BCCR2 (aa 1443–1648), and BCCR2′ (aa 1754–1854); for BRCA2, BCCR1 (aa 1–199), BCCR1′ (aa 258–602), OCCR1 (aa 1083–1894), OCCR2 (aa 2215–2491), and BCCR2 (aa 2465–2968).

### 2.6. Statistical Analysis

All inferential analyses were restricted to pathogenic mutations. The primary analysis tested whether the proportion of pathogenic mutations occurring in each protein domain differed between HR+/HER2− and TNBC subtypes, using Fisher’s exact test on 2 × 2 contingency tables for each domain. Odds ratios (ORs) and 95% confidence intervals were calculated using the Woolf method with continuity correction (0.5 added to each cell) when any cell count was zero. Multiple comparisons across the nine domain tests (four *BRCA1* domains, five *BRCA2* domains) were controlled using the Benjamini–Hochberg false discovery rate (BH-FDR) method, with adjusted *p*-values reported. Analogous tests were applied for the nine Rebbeck BCCR/OCCR cluster regions (four *BRCA1*: BCCR1, OCCR, BCCR2, and BCCR2′; five *BRCA2*: BCCR1, BCCR1′, OCCR1, OCCR2, and BCCR2), with FDR correction performed within the cluster-region family separately. Between-cohort heterogeneity for the principal contrast (*BRCA1* BRCT × subtype) was quantified by Cochran’s Q test and the I^2^ statistic. Pooled effect estimates across cohorts were computed using inverse-variance-weighted fixed-effects meta-analysis on the log-odds-ratio scale, with random effects (DerSimonian–Laird) sensitivity analysis where appropriate. To explicitly address the statistical power limitations of the available sample size, we performed two additional analyses. First, post hoc power analyses were conducted via Monte Carlo simulation (10,000 iterations per scenario, two-sided Fisher’s exact test, and α = 0.05) to estimate the minimum detectable effect (MDE) for the principal *BRCA1* BRCT × subtype contrast. Second, formal equivalence testing was conducted using the Two One-Sided Tests (TOSTs) procedure on the difference in mutation proportions, with prespecified equivalence margins of ±10%, ±15%, ±20%, ±25%, and ±30% absolute difference; equivalence within a margin was declared when the TOST *p*-value was less than α = 0.05 (i.e., both one-sided tests rejected). The ±20% margin was chosen a priori as a clinically meaningful threshold for declaring domain-level distributions “similar” in a context where decisions about *BRCA1/2* testing eligibility are not refined at the single-domain level. Statistical significance for primary analyses was prespecified as an FDR-adjusted *p* < 0.05. All analyses were performed in Python 3.11 using scipy 1.11. VUS variants were summarized descriptively by domain-level frequency with no inferential testing performed.

### 2.7. Use of Generative AI Tools

Generative artificial intelligence (Claude, Anthropic, model version Opus 4.7, accessed on 15 May 2026) was used to assist with the following aspects of manuscript preparation: (i) drafting and iterative editing of manuscript text based on author-provided study design, data, and analytical results; (ii) assisting in the formulation of Python and JavaScript code for data extraction from the cBioPortal REST API and for Fisher’s exact tests with Benjamini–Hochberg false discovery rate correction, with all code outputs reviewed and verified by the authors; (iii) systematic cross-checking of bibliographic references against PubMed records; and (iv) critical review of the manuscript from statistical, genetic, and clinical oncology perspectives. All data interpretation, scientific claims, statistical conclusions, and final editorial decisions were made by the authors. The authors have reviewed, verified, and edited all AI-assisted content and take full responsibility for the originality, validity, and integrity of the manuscript.

## 3. Results

### 3.1. Cohort Characteristics and Mutation Inventory

Across the three cohorts (TCGA-BRCA, METABRIC, and MSK-CHORD), a total of 394 somatic *BRCA1/2* mutations were identified in breast cancer patients with available receptor classification (Table 1). *BRCA1* accounted for 166 (42.1%) and *BRCA2* for 228 (57.9%) of all mutations. Of the 394 mutations, 147 (37.3%) met prespecified pathogenic criteria (nonsense, frameshift, or canonical splice site), while 247 (62.7%) were classified as VUSs (missense or in-frame insertions/deletions). The largest contributor to the dataset was the MSK-CHORD cohort (*n* = 282), reflecting both its greater overall sample size (25,040 patients) and the contemporary clinical-grade sequencing approach that captures both primary and metastatic tumors. The MSK-CHORD cohort provides 110 of the 147 pathogenic mutations in the combined dataset, representing a substantial expansion in pathogenic variant numbers relative to TCGA-BRCA (*n* = 13) and METABRIC (*n* = 24) alone.

### 3.2. Subtype Distribution of Pathogenic BRCA1/2 Mutations

Among the 147 pathogenic *BRCA1/2* mutations identified across the combined cohort, the distribution by molecular subtype demonstrated that pathogenic variants were not confined to triple-negative disease (Table 2). Notably, 84 of 131 pathogenic mutations classified into the principal subtype contrast (HR+/HER2− or TNBC) occurred in HR+/HER2− tumors (64.1%), while 47 (35.9%) occurred in TNBC. For *BRCA1* specifically, the distribution was nearly balanced between HR+/HER2− (*n* = 32) and TNBC (*n* = 30), while for *BRCA2*, HR+/HER2− predominated (*n* = 52) over TNBC (*n* = 17), consistent with the established association of *BRCA2* with hormone receptor-positive disease.

### 3.3. BRCA1 Domain Distribution Is Similar Between HR+/HER2− and TNBC

**Figure 1:**
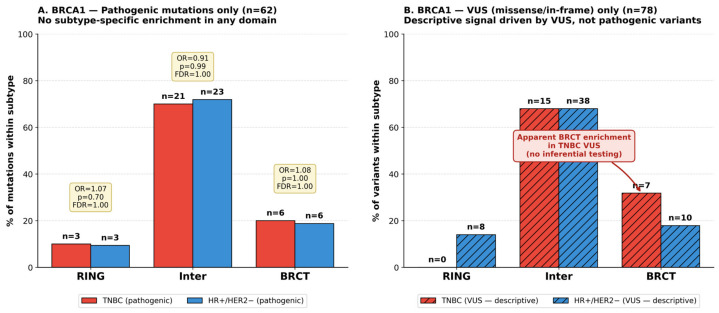
Pathogenic somatic *BRCA1/2* mutation distribution across functional protein domains by breast cancer subtype. (**A**) BRCA1 protein with RING (residues 1–109), inter-domain region (110–1645), and BRCT (1646–1855) domains shown to scale. (**B**) BRCA2 protein with N-terminal region (1–1001), eight BRC repeats (1002–2085), inter-DBD region (2086–2478), DNA-binding domain (DBD; 2479–3186), and C-terminal region (3187–3418). Each lollipop represents one pathogenic somatic mutation (nonsense, frameshift, or canonical splice site). Red lollipops above the protein backbone denote mutations from TNBC tumors; blue lollipops below the protein backbone denote mutations from HR+/HER2− tumors. Data are pooled across TCGA-BRCA, METABRIC, and MSK-CHORD cohorts (*n* = 131 pathogenic mutations in the principal subtype contrast). No domain demonstrated a statistically significant subtype-specific enrichment (all FDR-adjusted *p* = 1.00). Protein domain coordinates per UniProt (*BRCA1*: P38398; *BRCA2*: P51587). Yellow annotation boxes above each domain show the within-subtype proportion of pathogenic mutations falling in that domain (e.g., “TNBC 20% (6/30) · HR+ 19% (6/32)” for BRCT).

The spatial distribution of pathogenic *BRCA1* somatic mutations across functional protein domains, stratified by HR+/HER2− and TNBC subtypes, is illustrated in Figure 1A. The primary analysis tested whether the proportion of pathogenic *BRCA1* mutations falling within each functional protein domain differed between HR+/HER2− and TNBC subtypes (Table 3). Across 62 pathogenic *BRCA1* mutations in the principal subtype contrast, no domain showed a statistically significant subtype-specific enrichment. For the BRCT domain (the a priori domain of interest from our prior exploratory analysis), the frequency of pathogenic mutations was virtually identical between subtypes: 20.0% (6/30) in TNBC and 18.8% (6/32) in HR+/HER2− (OR = 1.08, 95% CI: 0.31–3.78; raw *p* = 1.00; and FDR-adjusted *p* = 1.00). Other domains showed similar near-null results: RING (TNBC 10.0% vs. HR+ 9.4%, OR = 1.07, and FDR-adjusted *p* = 1.00); inter-domain (TNBC 70.0% vs. HR+ 71.9%, OR = 0.91, and FDR-adjusted *p* = 1.00). The inter-domain region, encompassing the majority of the *BRCA1* coding sequence (residues 110–1645), accounted for the bulk of mutations in both subtypes, consistent with its size and with neutral mutation accumulation.

### 3.4. BRCA2 Domain Distribution Is Similar Between HR+/HER2− and TNBC

Analogous analyses for *BRCA2* (*n* = 69 pathogenic mutations in HR+/HER2− or TNBC) likewise showed no statistically significant subtype-specific domain enrichment (Figure 1B; Table 4). The DBD, which contains the principal DNA-binding apparatus essential for RAD51-mediated HR, demonstrated a numerical trend toward higher representation in HR+/HER2− (30.8%, 16/52) than TNBC (17.6%, 3/17; OR = 0.48, raw *p* = 0.36; and FDR-adjusted *p* = 1.00); however, this did not reach statistical significance. BRC repeats showed a modest non-significant trend in the opposite direction (TNBC 41.2% vs. HR+ 28.8%, OR = 1.73, and raw *p* = 0.38; FDR *p* = 1.00). Across all five *BRCA2* domains, no FDR-adjusted *p*-value reached the prespecified significance threshold.

### 3.5. Rebbeck BCCR/OCCR Cluster-Region Analysis

**Figure 2:**
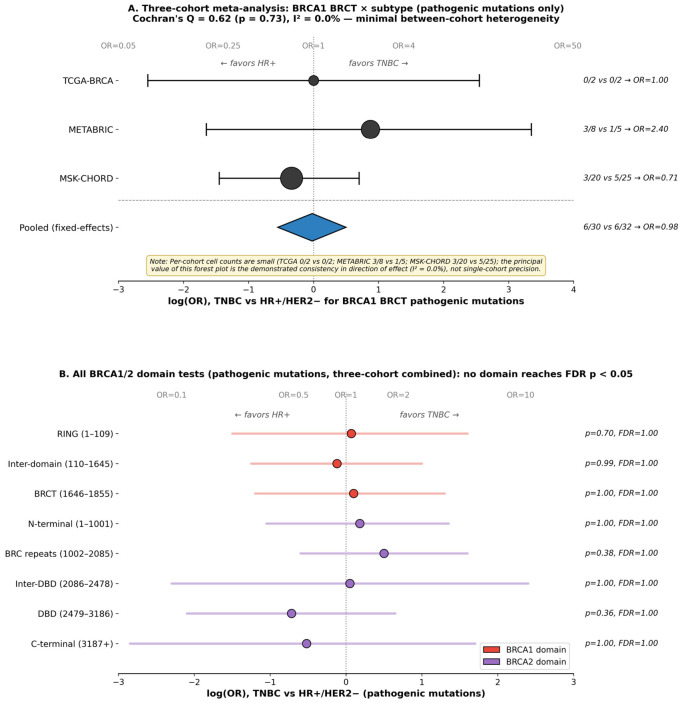
Three-cohort meta-analysis and domain-level odds-ratio landscape for pathogenic *BRCA1/2* mutations. (**A**) Forest plot of the principal *BRCA1* BRCT × subtype contrast across TCGA-BRCA, METABRIC, and MSK-CHORD cohorts. Per-cohort log-odds ratios are shown with 95% confidence intervals; marker size is proportional to cohort weight. The diamond denotes the inverse-variance fixed-effects pooled estimate. Cochran’s Q = 0.62 (df = 2, *p* = 0.73), I^2^ = 0.0%, indicating minimal between-cohort heterogeneity and consistent null findings. (**B**) Aggregate landscape of all *BRCA1* and BRCA2 protein domains tested for subtype-specific enrichment in the pathogenic-mutation analysis. None of the nine domain tests reached statistical significance after Benjamini–Hochberg FDR correction. *BRCA1* domains shown in pink; *BRCA2* domains in purple.

To complement the UniProt domain-based analysis and to facilitate direct comparison with the seminal germline mutation location literature, we additionally examined the Rebbeck-defined breast cancer cluster regions (BCCRs) and ovarian cancer cluster regions (OCCRs) in our somatic mutation dataset (Table 5). The complete cluster-region set comprised four BRCA1 regions (BCCR1, OCCR, BCCR2, and BCCR2′) and five BRCA2 regions (BCCR1, BCCR1′, OCCR1, OCCR2, and BCCR2), totaling nine cluster-region tests. For BRCA1, the OCCR (residues 460–1354, encompassing the central exon 11 region) contained the largest fraction of mutations in both subtypes (TNBC 40.0% vs. HR+ 28.1%, OR = 1.70, and raw *p* = 0.42; FDR *p* = 1.00). BCCR1 (RING-adjacent, aa 60–169) showed slightly higher representation in TNBC (13.3% vs. 9.4%; OR = 1.49, NS), and BCCR2/BCCR2′ showed a near-balanced representation between subtypes. For *BRCA2*, OCCR1 (BRC-repeat-spanning, aa 1083–1894) showed a non-significant trend toward TNBC enrichment (35.3% vs. 21.2%, OR = 2.03, and raw *p* = 0.33), while BCCR2 (DBD-spanning, aa 2465–2968) showed the opposite trend (TNBC 11.8% vs. HR+ 23.1%, OR = 0.44, and raw *p* = 0.49). After FDR correction across all nine cluster-region tests, no cluster region reached statistical significance. The cumulative landscape of all domain-level odds ratios, depicted in Figure 2B, illustrates the null character of these contrasts across both genes. These findings indicate that the established germline BCCR/OCCR landscape, which is calibrated against breast-versus-ovarian cancer risk, does not translate into a corresponding HR+ versus TNBC subtype-specific enrichment for somatic mutations in breast cancer.

### 3.6. Cohort Heterogeneity Assessment

To assess whether the null findings were consistent across cohorts or driven by between-cohort heterogeneity, we computed Cochran’s Q and I^2^ statistics for the principal *BRCA1* BRCT × subtype contrast (Figure 2A; Table 6). Across the three cohorts, the log-odds ratios were highly consistent (TCGA: log OR = 0.00; METABRIC: log OR = 0.88; and MSK-CHORD: log OR = −0.35), and Cochran’s Q was 0.62 (df = 2, *p* = 0.73), corresponding to an I^2^ of 0.0%. Inverse-variance fixed-effects meta-analysis produced a pooled OR of 0.98, with all three cohorts contributing concordant near-null estimates. This minimal heterogeneity is in contrast to our prior preliminary analysis based only on TCGA-BRCA and METABRIC, which had yielded a positive BRCT signal (OR = 5.33, raw *p* = 0.007); that signal was driven by the inclusion of VUS missense variants in the analytic set, as detailed in the following section.

### 3.7. Descriptive VUS Distribution (Excluded from Inferential Analyses)

**Figure 3:**
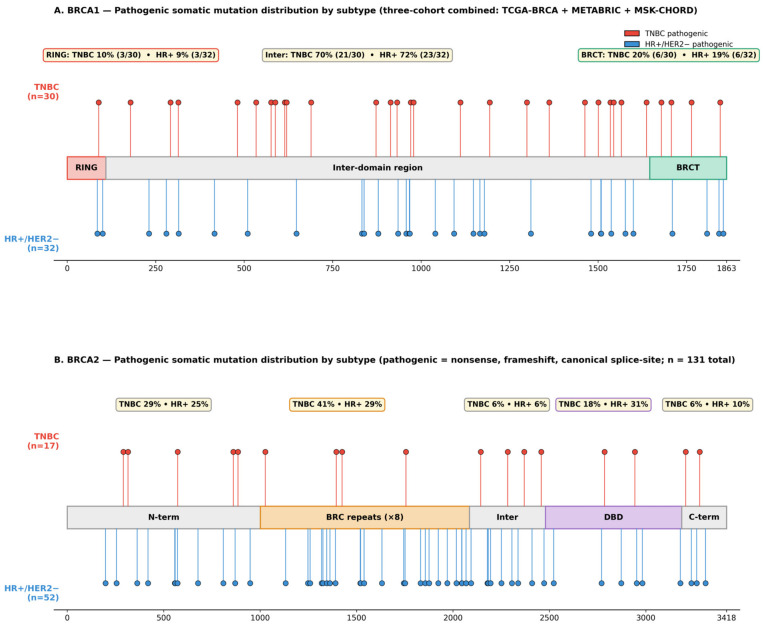
Methodological lesson: apparent BRCT enrichment in unfiltered analyses is driven by VUSs, not pathogenic variants. Comparison of *BRCA1* domain-level distributions in TNBC versus HR+/HER2− subtypes when stratified by variant class. (**A**) Pathogenic-only analysis (*n* = 62): the *BRCA1* BRCT domain contains a virtually identical fraction of mutations in TNBC (20.0%) and HR+/HER2− (18.8%); OR = 1.08, FDR-adjusted *p* = 1.00. (**B**) VUS-only descriptive analysis (*n* = 78, missense and in-frame variants only, no inferential testing performed): the *BRCA1* BRCT domain shows a higher fraction in TNBC (31.8%) than HR+/HER2− (17.9%). Pooling pathogenic and VUS variants together produces apparent BRCT enrichment that does not extend to the pathogenic, clinically actionable variant subset. This illustrates why prespecified pathogenicity filtering is essential for clinically informative inference. Per ACMG and ASCO guidance, VUSs should not be incorporated into PARP inhibitor decision-making.

For completeness and to inform future functional studies, we report the descriptive distribution of VUSs (missense and in-frame variants) by domain and subtype, with the explicit caveat that no inferential testing was performed on this dataset (Table 7; Figure 3). Among 78 *BRCA1* VUSs in the principal subtype contrast (TNBC *n* = 22; HR+/HER2− *n* = 56), the BRCT domain contained a numerically larger fraction in TNBC (31.8%, 7/22) than in HR+/HER2− (17.9%, 10/56). This is consistent with the apparent BRCT enrichment signal observed when pathogenic and VUS variants are pooled together in unfiltered analyses. By contrast, the corresponding pathogenic-only analysis showed no enrichment (TNBC 20.0% vs. HR+ 18.8%; Figure 3A versus Figure 3B). The disparity between these two analyses—a notable VUS signal that does not extend to pathogenic mutations—illustrates the methodological hazard of unfiltered domain enrichment analysis and supports the prespecified analytical separation of pathogenic and VUS classes. For transparent variant-level reporting, all 17 BRCT-localized VUS variants (TNBC *n* = 7; HR+/HER2− *n* = 10) are listed individually with their subtype, cohort, and amino acid position in Supplementary Table S1.

For *BRCA2* VUSs (*n* = 126 in HR+/HER2− or TNBC), the descriptive domain distribution was approximately uniform across subtypes for all five domains (TNBC vs. HR+ percentages: N-terminal 29.6% vs. 32.3%; BRC repeats 18.5% vs. 21.2%; inter-DBD 18.5% vs. 12.1%; DBD 22.2% vs. 21.2%; and C-terminal 11.1% vs. 13.1%) without the BRCT-like discrepancy seen for *BRCA1*.

### 3.8. Statistical Power and Equivalence Testing

To explicitly address the statistical power limitations of our sample size, we performed post hoc power analyses and formal equivalence testing for the principal *BRCA1* BRCT × subtype contrast (Table 8). Monte Carlo simulation (10,000 iterations per scenario; two-sided Fisher’s exact test, α = 0.05) indicated that at the available sample sizes (*n* = 30 TNBC and *n* = 32 HR+ pathogenic *BRCA1* mutations), the study had approximately 80% power to detect only large effects, corresponding to a TNBC mutation rate of approximately 54% versus the observed HR+ rate of 18.8% (OR ≈ 5.0). Smaller effects had substantially lower power: OR = 3.0 yielded approximately 47% power, and OR = 1.7 (a typical “modest” enrichment) yielded only about 12% power. Two One-Sided Tests (TOSTs) for differences in proportions were performed at prespecified equivalence margins of ±10%, ±15%, ±20%, ±25%, and ±30% absolute difference. For the *BRCA1* BRCT contrast (observed difference = +1.3 percentage points), equivalence was statistically supported at the ±20% margin (TOST *p* = 0.031), ±25% margin (TOST *p* = 0.009), and ±30% margin (TOST *p* = 0.002); equivalence at tighter margins (±10% and ±15%) was not supported by the data. By contrast, for the BRCA2 DBD contrast (observed difference = −13.1 percentage points; OR = 0.48 in the HR+/HER2− direction), TOSTs failed to support the equivalence at any margin up to ±30%, indicating that this trend cannot be statistically dismissed and warrants prospective investigation in larger somatic cohorts. We therefore frame our principal domain-level findings as “no evidence of large subtype-specific enrichment” rather than as positive evidence of biological equivalence, and we explicitly note that the previously hypothesized BRCT enrichment of OR ≈ 5.3 (observed in our preliminary unfiltered two-cohort analysis) is excluded by the upper bound of the present 95% confidence interval (OR upper bound = 3.78).

## 4. Discussion

In this three-cohort analysis comprising 394 somatic *BRCA1/2* mutations from 28,366 breast cancer samples (TCGA-BRCA, METABRIC, and MSK-CHORD), we did not detect statistically significant subtype-specific enrichment of pathogenic mutations in any functional protein domain of *BRCA1* or *BRCA2* nor in the Rebbeck-defined breast or ovarian cancer cluster regions. Importantly, however, the absence of statistical significance should not be interpreted as positive evidence of biological equivalence. Post hoc power analysis indicated that the available sample size (*n* = 62 *BRCA1* and *n* = 69 *BRCA2* pathogenic mutations in HR+/HER2− and TNBC) only supports the reliable detection of large effects (OR ≥ ~3.0 for the principal *BRCA1* BRCT × subtype contrast); small to moderate effects cannot be excluded. Three principal observations may be drawn within these limits. First, pathogenic mutations were broadly distributed across all *BRCA1* domains (RING, inter-domain, and BRCT) and *BRCA2* domains (N-terminal, BRC repeats, inter-DBD, DBD, and C-terminal) in both subtypes, with all nine FDR-adjusted *p*-values equal to 1.00 (Table 3 and Table 4). For the principal *BRCA1* BRCT contrast, formal equivalence testing (TOST) demonstrated equivalence within a prespecified ±20% margin (TOST *p* = 0.031), although tighter margins (±15%) were not supported by the data. Second, the majority (64.1%) of pathogenic *BRCA1/2* mutations occurred in HR+/HER2− disease rather than TNBC. Third, the absence of large subtype-specific enrichment was highly consistent across the three independent cohorts (Cochran’s Q = 0.62, I^2^ = 0.0%; Table 6), with a pooled inverse-variance OR of 0.98 for the principal *BRCA1* BRCT × subtype contrast.

Our findings warrant interpretation in the context of prior literature on the BRCA1/2 mutation location and cancer phenotype. The germline mutation literature, particularly the CIMBA-based analyses by Rebbeck and colleagues, has firmly established that mutation position influences the relative risk of breast versus ovarian cancer—the BCCR and OCCR phenomenon—and these cluster regions remain biologically and clinically meaningful for germline BRCA carriers [18]. Our somatic mutation data, examined within breast cancers alone, do not extend this BCCR/OCCR framework to subtype-specific (HR+ vs. TNBC) differentiation, although the limited sample size constrains the precision of this null observation. This is biologically plausible: the BCCR/OCCR framework was calibrated against organ-level risk (which organ develops cancer in a carrier) not against the within-organ phenotypic subtype that emerges once a cancer has arisen. The mutation localization that biases an unaffected BRCA carrier toward breast versus ovarian malignancy is mechanistically distinct from the subtype that a breast cancer takes once mutational and lineage commitment events have occurred.

A central methodological observation of our study concerns the marked difference between unfiltered analyses (pooling pathogenic and VUS variants) and pathogenicity-filtered analyses. An unpublished preliminary two-cohort analysis by our group (TCGA-BRCA and METABRIC only, with all somatic *BRCA1/2* mutations regardless of variant class) had suggested an apparent enrichment of *BRCA1* BRCT mutations in TNBC (OR ≈ 5.3, raw *p* = 0.007). In the present larger, three-cohort, pathogenicity-restricted dataset, that signal is absent (OR = 1.08 for BRCT; 95% CI 0.31–3.78, which excludes the previously observed ~5-fold effect at the upper bound). Descriptive examination revealed that the original signal was driven by *BRCA1* missense (VUS) variants, which were over-represented in the BRCT region of TNBC samples (31.8% vs. 17.9% in HR+) without an accompanying pathogenic signal. Several explanations may apply: BRCT missense variants may accumulate as functionally silent passenger events; receptor-defined subtypes may differ in baseline mutational pressure; or the apparent VUS difference may reflect statistical fluctuation in heterogeneous variant categories. Whichever explanation applies, the principal implication is that domain-level analyses that do not separate pathogenic from VUS variants can produce signals that do not extend to the clinically actionable subset of mutations. This concords with the prior literature emphasizing that VUSs lack the functional certainty required for clinical decision-making [21,22] and supports the routine practice in cancer genomics studies of separating these variant classes during analysis [23,35].

From a clinical perspective, three implications emerge, presented with appropriate moderation. First, pathogenic *BRCA1/2* somatic mutations are not restricted to TNBC; in our combined dataset, 64.1% occurred in HR+/HER2− disease. This observation parallels and extends earlier germline data showing that pathogenic *BRCA1/2* prevalence in HR+ breast cancer is non-trivial and clinically meaningful [11,12]. We acknowledge that broader BRCA testing eligibility across breast cancer subtypes is already reflected in current NCCN, ESMO, and ASCO recommendations [6,7,12], and the principal value of our somatic data is therefore as cohort-scale supporting evidence for this existing consensus rather than as a practice-changing finding. Second, within the statistical power available, intragenic *BRCA1/2* domain localization—as inferred from somatic mutations—did not emerge as a subtype-specific biomarker in our analyses. Decisions about PARP inhibitor candidacy should continue to rely on the binary pathogenic-versus-non-pathogenic classification, augmented where appropriate by homologous recombination deficiency scoring or related genomic assays [3,4,36], rather than on intragenic location. Third, the dichotomy between VUS and pathogenic signals in our data reinforces the existing clinical guidance that VUSs should not be incorporated into PARP inhibitor decision-making [21,22]; from an analytical perspective, this dichotomy also indicates that domain-level studies should prespecify variant filtering.

The clinical context of PARP inhibitor use deserves brief comment. PARP inhibitors are approved for the treatment of HER2-negative breast cancer with germline BRCA1/2 pathogenic variants based on the OlympiAD and EMBRACA trials, which demonstrated progression-free survival benefit with olaparib and talazoparib, respectively [3,4]. The OlympiA trial extended olaparib to the adjuvant setting in patients with high-risk early breast cancer harboring germline BRCA1/2 pathogenic variants, again enrolling across receptor subtypes [20,37]. Our finding that the intragenic location of pathogenic mutations does not differ measurably by subtype is consistent with the clinical observation that PARP inhibitor activity tracks with pathogenic mutation status rather than with where in the gene the mutation occurs. Future characterization of BRCA1/2 biology in breast cancer is likely to benefit from technologies that extend beyond conventional short-read sequencing, including optical genome mapping for large structural rearrangements [38] and integrated multi-omics approaches that combine genomic, transcriptomic, and proteomic data [39]. Likewise, treatment response in BRCA-mutant tumors is increasingly recognized to depend not only on mutation status but also on broader genomic context, acquired resistance, and immune-related factors; exploratory combinations with immune checkpoint inhibition in selected BRCA-mutant tumor settings [40] illustrate this evolving landscape and represent a direction for future investigation beyond simple mutation classification.

The strengths of this study include the use of three large, independent cohorts representing complementary methodological approaches (whole-exome, targeted-panel, and clinical-grade matched tumor/normal sequencing); a substantial increase in pathogenic mutation numbers (*n* = 147) relative to prior analyses; prespecified pathogenicity filtering aligned with ENIGMA/ACMG guidelines and clinical practice; explicit separation of inferential analyses (pathogenic variants) from descriptive analyses (VUS); rigorous statistical methodology including FDR correction, formal between-cohort heterogeneity assessment, post hoc power analysis, and formal equivalence testing; and transparent reporting of the relationship between the present pathogenicity-filtered analysis and a prior preliminary unfiltered analysis. We note, however, that the principal analytical framework is built on Fisher’s exact tests for predefined domain × subtype contingencies; it characterizes mutation distribution patterns rather than biological function or treatment response, and the methodological rigor should not be confused with biological novelty.

Several limitations warrant explicit acknowledgment. First, and most fundamentally, the available sample size is modest given the multiplicity of domain and cluster-region tests, and our null findings should be interpreted as “no evidence of large subtype-specific enrichment” rather than as positive evidence of biological equivalence. As a concrete example, for the BRCA1 BRCT × subtype contrast (6 of 30 TNBC vs. 6 of 32 HR+/HER2−), the 95% confidence interval for the odds ratio (0.31–3.78) is compatible with anything from a roughly 3-fold depletion to a roughly 4-fold enrichment in TNBC; modest subtype-specific effects could exist but be undetectable at the current sample size. Second, although none of the BRCA2 domain contrasts reached statistical significance, the BRCA2 DBD trend (TNBC 17.6% vs. HR+ 30.8%, OR = 0.48) is biologically intriguing given the DBD’s central role in RAD51-mediated homologous recombination and BRCA2’s established association with HR+ disease; the opposite trend for the BRC repeats (TNBC 41.2% vs. HR+ 28.8%) is similarly notable. Equivalence testing did not support equivalence for the DBD contrast at the ±20% margin, and these trends warrant prospective investigation in substantially larger somatic cohorts. Third, our analyses are restricted to somatic mutation data; germline-annotated cohorts of comparable size with subtype information and somatic mutation profiling are not currently available. The relationship between germline and somatic BRCA1/2 mutation localization within the same breast tumor is an important question requiring datasets that link both. Fourth, variant classification was operationalized as truncating-versus-non-truncating, a conservative surrogate for ACMG-grade pathogenicity classification. While truncating mutations are reliably loss-of-function, well-characterized missense pathogenic variants (e.g., in BRCT) may be misclassified as a VUS; their small numbers in somatic datasets make this unlikely to alter principal conclusions. Fifth, the three cohorts differ in sequencing approach—TCGA-BRCA used whole-exome sequencing with a matched normal; METABRIC used targeted Agilent SureSelect (173 genes); and MSK-CHORD used MSK-IMPACT (468 genes) with matched tumor/normal pairs, allowing for the confident exclusion of germline variants [31,32]. All three platforms fully cover BRCA1/2 coding regions, but ascertainment sensitivity may differ for indels, splice variants beyond canonical positions, and large structural rearrangements; the last of these is largely invisible to all three panels and is the subject of complementary methods such as optical genome mapping [38]. Sixth, MSK-CHORD contributes the majority of pathogenic mutations (110 of 147) and enrichments for metastatic and advanced disease; this could introduce stage-related differences in mutational pressure across cohorts, although the very low observed cohort heterogeneity (I^2^ = 0.0%) argues against this being a major confounder for the principal contrast. We are not aware of patient overlap between the three cohorts, which are institutionally and temporally distinct. Finally, our analyses examine domain-level distributions and do not capture subdomain (e.g., residue-level hot spot) patterns that might require substantially larger datasets to detect.

In conclusion, within the statistical power available, our three-cohort analysis shows no evidence of large subtype-specific enrichment of pathogenic *BRCA1/2* somatic mutations across protein domains or Rebbeck-defined cluster regions in HR+/HER2− and TNBC breast cancer; small to moderate effects cannot be excluded with the present sample size. The majority of pathogenic mutations occur in HR+/HER2− disease, providing cohort-scale supporting evidence for current guidelines that recommend *BRCA1/2* testing across breast cancer subtypes. The most novel methodological contribution of this work is the demonstration that the apparent domain-level enrichment seen in prior unfiltered analyses can reflect VUSs rather than pathogenic variants, underscoring the importance of prespecified pathogenicity filtering for clinically informative inference. Larger somatic datasets, ideally paired with germline status and structural-variant-aware sequencing technologies, will be needed to determine whether the modest *BRCA2* DBD and BRC-repeat trends observed here reflect true subtype-specific biology.

## Figures and Tables

**Table 1:** Cohort characteristics and *BRCA1/2* mutation inventory.

Cohort	Total Samples	*BRCA1* Mut.	*BRCA2* Mut.	Pathogenic (*n*)	VUS (*n*)
TCGA-BRCA	817	17	20	13	24
METABRIC	2509	38	37	24	51
MSK-CHORD	25,040	111	171	110	172
Combined	28,366	166	228	147	247

Note: METABRIC samples with incomplete receptor information were excluded from inferential analyses. Mutation counts in the table refer to total mutations per gene (some patients carried more than one mutation). Pathogenic = truncating (nonsense, frameshift, and canonical splice site); VUS = missense or in-frame.

**Table 2:** Cross-tabulation of pathogenic *BRCA1/2* mutations by gene and breast cancer subtype (three-cohort combined).

Subtype	*BRCA1* Pathogenic	*BRCA2* Pathogenic	Total	% of Pathogenic
HR+/HER2−	32	52	84	64.1%
TNBC	30	17	47	35.9%
HR+/HER2+	3	7	10	—
HR−/HER2+	3	3	6	—
Total (HR+/HER2− + TNBC)	62	69	131	100%

**Table 3:** BRCA1 protein domain distribution of pathogenic somatic mutations by subtype (three-cohort combined; *n* = 62).

Domain	TNBC *n*	HR+ *n*	% TNBC	% HR+	OR	FDR *p*
RING (1–109)	3	3	10.0%	9.4%	1.07	1.00
Inter-domain (110–1645)	21	23	70.0%	71.9%	0.91	1.00
BRCT (1646–1855)	6	6	20.0%	18.8%	1.08	1.00
Total	30	32	100%	100%	—	—

OR = odds ratio for the domain in TNBC vs. HR+/HER2−. FDR *p* = Benjamini–Hochberg-adjusted *p*-value across 9 domain tests (4 *BRCA1* + 5 *BRCA2*). No domain reached statistical significance after FDR correction.

**Table 4:** BRCA2 protein domain distribution of pathogenic somatic mutations by subtype (three-cohort combined; *n* = 69).

Domain	TNBC *n*	HR+ *n*	% TNBC	% HR+	OR	FDR *p*
N-terminal (1–1001)	5	13	29.4%	25.0%	1.25	1.00
BRC repeats (1002–2085)	7	15	41.2%	28.8%	1.73	1.00
Inter-DBD (2086–2478)	1	3	5.9%	5.8%	1.02	1.00
DBD (2479–3186)	3	16	17.6%	30.8%	0.48	1.00
C-terminal (3187+)	1	5	5.9%	9.6%	0.59	1.00
Total	17	52	100%	100%	—	—

**Table 5:** Rebbeck BCCR/OCCR cluster-region distribution of pathogenic somatic *BRCA1/2* mutations by subtype (three-cohort combined; *n* = 131; and 9 cluster-region tests).

Gene	Cluster Region	Coordinates (aa)	TNBC *n*	HR+ *n*	% TNBC	% HR+	OR	Raw *p*	FDR *p*
*BRCA1*	BCCR1	60–169	4	3	13.3%	9.4%	1.49	0.70	1.00
*BRCA1*	OCCR	460–1354	12	9	40.0%	28.1%	1.70	0.42	1.00
*BRCA1*	BCCR2	1443–1648	5	5	16.7%	15.6%	1.08	1.00	1.00
*BRCA1*	BCCR2′	1754–1854	2	2	6.7%	6.2%	1.07	1.00	1.00
*BRCA2*	BCCR1	1–199	0	2	0.0%	3.8%	0.00 *	1.00	1.00
*BRCA2*	BCCR1′	258–602	3	7	17.6%	13.5%	1.38	0.70	1.00
*BRCA2*	OCCR1	1083–1894	6	11	35.3%	21.2%	2.03	0.33	1.00
*BRCA2*	OCCR2	2215–2491	1	1	5.9%	1.9%	3.19	0.43	1.00
*BRCA2*	BCCR2	2465–2968	2	12	11.8%	23.1%	0.44	0.49	1.00

OR = odds ratio for the cluster region in TNBC vs. HR+/HER2−. Raw *p* = two-sided Fisher’s exact test. FDR *p* = Benjamini–Hochberg-adjusted *p*-value across the 9 cluster-region tests. * Continuity correction applied (zero-cell). No cluster region reached statistical significance after FDR correction. Cluster-region coordinates per Rebbeck et al. (2015), JAMA [18], converted from cDNA to amino-acid coordinates.

**Table 6:** Cohort-level effect estimates and heterogeneity assessment (pathogenic *BRCA1* BRCT × subtype).

Cohort	BRCT TNBC/ Total TNBC	BRCT HR+/ Total HR+	Cohort OR	log(OR)
TCGA-BRCA	0/2	0/2	1.00	0.00
METABRIC	3/8	1/5	2.40	+0.88
MSK-CHORD	3/20	5/25	0.71	−0.35
Pooled (fixed effects)	6/30	6/32	0.98	−0.02

Cochran’s Q = 0.62 (df = 2, *p* = 0.73); I^2^ = 0.0% (minimal heterogeneity).

**Table 7:** Descriptive distribution of *BRCA1* VUSs (missense and in-frame variants) by domain and subtype (no inferential testing performed).

Domain	TNBC *n*	% TNBC	HR+/HER2− *n*	% HR+
RING (1–109)	0	0.0%	8	14.3%
Inter-domain (110–1645)	15	68.2%	38	67.9%
BRCT (1646–1855)	7	31.8%	10	17.9%
Total VUSs	22	100%	56	100%

Note: VUSs = variants of uncertain significance (missense, in-frame insertion/deletion). Per ACMG/ASCO guidance, VUSs are not used in clinical decision-making and were excluded from inferential analyses in this study.

**Table 8:** Post hoc statistical power and TOST equivalence testing for the principal *BRCA1* BRCT × subtype contrast (pathogenic mutations; *n* = 62).

Analysis	Parameter	Value
Sample size	TNBC/HR+ pathogenic *BRCA1*	*n* = 30/*n* = 32
Observed BRCT	TNBC/HR+ mutation rate	20.0% (6/30)/18.8% (6/32)
Observed effect	OR (95% CI)	1.08 (0.31–3.78)
	Absolute difference	+1.3 percentage points
Power (Monte Carlo)	Detectable OR at 80% power	≈5.0 (TNBC ≥ ~54%)
	Power at OR = 3.0	~47%
	Power at OR = 1.7	~12%
TOST equivalence	Margin ±10%	TOST *p* = 0.192 (not equivalent)
	Margin ±15%	TOST *p* = 0.086 (not equivalent)
	Margin ±20%	TOST *p* = 0.031 (EQUIVALENT)
	Margin ±25%	TOST *p* = 0.009 (equivalent)
	Margin ±30%	TOST *p* = 0.002 (equivalent)

Power estimated by Monte Carlo simulation (10,000 iterations per scenario) using two-sided Fisher’s exact test at α = 0.05. TOST = Two One-Sided Tests for equivalence of two proportions. Equivalence is declared when both one-sided null hypotheses (lower bound and upper bound of the prespecified margin) are rejected at α = 0.05.

## Data Availability

All mutation and clinical data analyzed in this study are publicly available via the cBioPortal for Cancer Genomics (https://www.cbioportal.org). The specific datasets used were: TCGA-BRCA (study ID: brca_tcga_pub2015; reference: Ciriello et al., Cell 2015, DOI: 10.1016/j.cell.2015.09.033), METABRIC (study ID: brca_metabric; references: Curtis et al., Nature 2012, DOI: 10.1038/nature10983 and Pereira et al., Nat Commun 2016, DOI: 10.1038/ncomms11479), and MSK-CHORD (study ID: msk_chord_2024; Nature 2024). Analysis scripts and derived data files used in this manuscript are available from the corresponding author upon reasonable request.

## Supplementary Materials

The following supporting information can be downloaded at: https://www.mdpi.com/article/10.3390/genes17060693/s1, Table S1: Individual *BRCA1* BRCT-domain VUS Variants by Subtype and Cohort.
