# Supplementary material for: Domain-Level Distribution of Pathogenic BRCA1/2 Somatic Mutations Shows No Evidence of Large Subtype-Specific Enrichment in Breast Cancer: A Three-Cohort Analysis Supporting Broad BRCA Testing
Source: Genes (Basel). 2026 Jun 13;17(6):693. doi: 10.3390/genes17060693 (PMC13299929; doi:10.3390/genes17060693)
Supplement: Supplementary file 1 [file genes-17-00693-s001.zip › genes-4345781-supplementary.pdf]

Supplementary Table S1

Individual BRCA1 BRCT-domain VUS Variants by Subtype and Cohort

All BRCA1 BRCT-domain variants of uncertain significance (VUS; missense and in-frame insertions/deletions) identified across the three combined cohorts (TCGA-BRCA, METABRIC, MSK-CHORD) in HR+/HER2− and TNBC subtypes. BRCT domain coordinates: amino acid positions 1646–1855 (UniProt P38398). Total n = 17 (TNBC n = 7; HR+/HER2− n = 10). This table supports the descriptive finding in Figure 3B that BRCT VUS are more frequently observed in TNBC samples (31.8% of all BRCA1 VUS in TNBC) than in HR+/HER2− samples (17.9%), despite no enrichment of pathogenic mutations in the same domain.

#	Subtype	Cohort	Amino Acid Position	Variant Type
1	**HR+/HER2-**	MSK-CHORD	p.1678	Missense
2	**HR+/HER2-**	MSK-CHORD	p.1685	Missense
3	**HR+/HER2-**	MSK-CHORD	p.1705	Missense
4	**HR+/HER2-**	MSK-CHORD	p.1795	Missense
5	**HR+/HER2-**	TCGA-BRCA	p.1811	Missense
6	**HR+/HER2-**	MSK-CHORD	p.1817	Missense
7	**HR+/HER2-**	METABRIC	p.1827	Missense
8	**HR+/HER2-**	MSK-CHORD	p.1835	Missense
9	**HR+/HER2-**	MSK-CHORD	p.1836	Missense
10	**HR+/HER2-**	MSK-CHORD	p.1837	Missense
11	**TNBC**	MSK-CHORD	p.1688	InFrameDel
12	**TNBC**	METABRIC	p.1692	Missense
13	**TNBC**	MSK-CHORD	p.1709	Missense
14	**TNBC**	MSK-CHORD	p.1781	Missense
15	**TNBC**	TCGA-BRCA	p.1788	Missense
16	**TNBC**	MSK-CHORD	p.1801	Missense
17	**TNBC**	METABRIC	p.1836	Missense

Notes:

Variant nomenclature uses simplified amino acid position (p.X) due to heterogeneous HGVS annotation across the source datasets via cBioPortal. Variant types per cBioPortal classification. None of the variants listed met inferential pathogenicity criteria (nonsense, frameshift, or canonical splice-site); all are reported as descriptive data only and are not used for clinical decision-making per ACMG/ASCO guidelines (References 21, 22 in main manuscript).
